# Rapidly Deteriorating Degenerative Cervical Myelopathy Following Ventricular Shunt Revision for Hydrocephalus: Case Report

**DOI:** 10.2196/48222

**Published:** 2023-08-28

**Authors:** Tanzil Rujeedawa, Oliver Mowforth, Mark Kotter, Benjamin Davies

**Affiliations:** 1 Division of Neurosurgery Department of Clinical Neurosciences University of Cambridge Cambridge United Kingdom

**Keywords:** cervical myelopathy, ossification of posterior longitudinal ligament, spondylosis, disk herniation, stenosis, spine, spinal, neck, disk, myelopathy, case, cervical, woman, women, ligament, gait

## Abstract

A female patient in her early 40s presented with a several-month history of gait unsteadiness and dragging her left leg. She had a background of congenital hydrocephalus, treated with a ventriculoatrial shunt. On examination, she had increased tone and brisk reflexes in the lower limbs and a positive Hoffmann sign. A computed tomography (CT) scan and shunt series x-rays identified hydrocephalus secondary to a disconnected shunt. Magnetic resonance imaging (MRI) of her cervical spine was also performed as part of the workup for her presenting symptoms and demonstrated features compatible with degenerative cervical myelopathy (DCM). The patient subsequently underwent a shunt revision. Following the operation, her walking and hand function deteriorated over a period of several weeks. She consequently underwent an anterior cervical decompression and fusion for DCM, which partially improved her symptoms.

The sequence of events suggests that the shunt surgery may have precipitated a worsening of the DCM. Possible explanations include spinal cord injury related to neck extension or hypoperfusion during intubation and general anesthesia or the loss of cerebrospinal fluid cushioning following the reinstitution of effective cerebrospinal fluid shunting. Surgeons should be alert to this possibility and offer prompt surgical intervention for DCM if required.

## Introduction

Degenerative cervical myelopathy (DCM) is the umbrella term for a range of chronic spinal injuries caused by cervical stenosis due to degenerative or congenital pathology [1,2]. DCM presents with motor or sensory dysfunction in the upper or lower limbs, such as the loss of dexterity, paresthesia and imbalance, pain, and bladder and bowel dysfunction [3]. We report the case of a patient with worsening DCM following the treatment of coexisting hydrocephalus.

## Case Report

A previously independent, self-employed female patient in her early 40s with congenital hydrocephalus presented with a dragging left leg and an abnormal gait for several months. There were no upper limb symptoms. On examination, there was increased tone and brisk reflexes in the lower limbs and a positive Hoffmann sign. Power and sensation were normal. She had an intracranial ventriculoatrial shunt, first inserted at 8 months old with 3 subsequent revisions. It was last revised when she was 15 years old. She is a married housekeeper with depression and anxiety, has never smoked, and does not drink alcohol.

**Figure 1:**
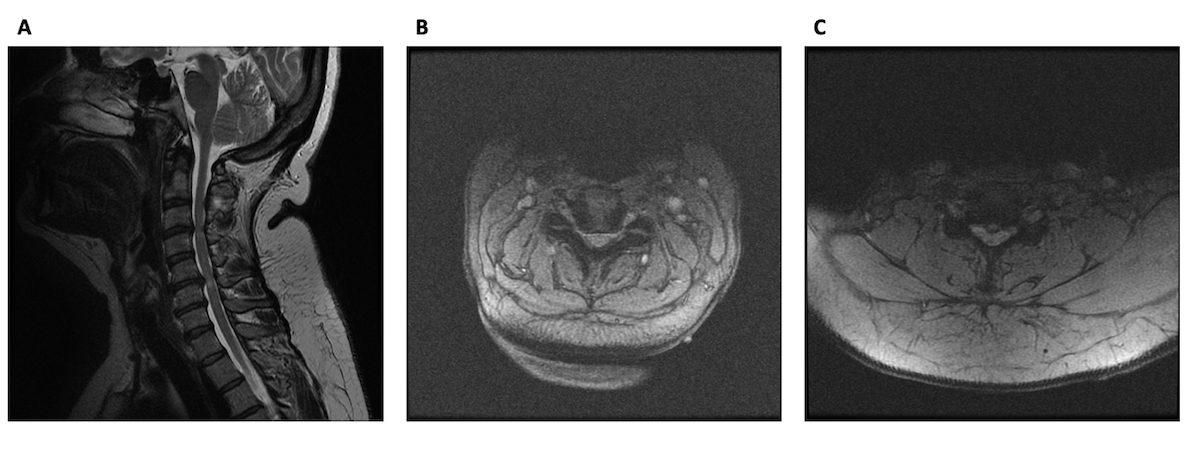
Investigations supporting a diagnosis of degenerative cervical myelopathy: (A) sagittal T2 MRI; (B) C3/4 axial T2 MRI; and (C) C5/6 axial T2 MRI. The MRI was performed 1 month before shunt revision. MRI: magnetic resonance imaging.

**Figure 2:**
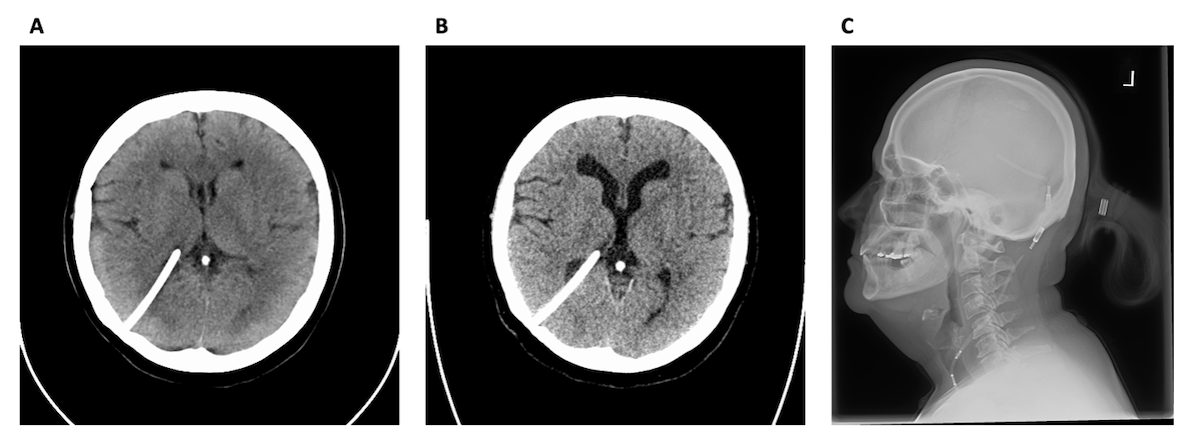
Investigations supporting hydrocephalus and shunt dysfunction: (A) CT scan from 13 years before the revision; (B) CT scan 1 month before the revision; and (C) shunt series x-ray 1 month before the revision. CT: computed tomography.

Magnetic resonance imaging (MRI) of her cervical spine demonstrated cord compression at C3/4 and C5/6 with T2 signal hyperintensity within the spinal cord (Figure 1), confirming a diagnosis of DCM. However, the shunt series x-rays also demonstrated the disconnection of the distal shunt catheter at the level of the external auditory meatus (Figure 2 C). A computed tomography (CT) head scan demonstrated that the lateral and third ventricles were minimally enlarged compared to the most recent CT scan performed less than 1 year earlier and were dilated compared to a previous scan 13 years earlier (Figures 2 A and B). There was no periventricular interstitial oedema or sulcal effacement.

**Figure 3:**
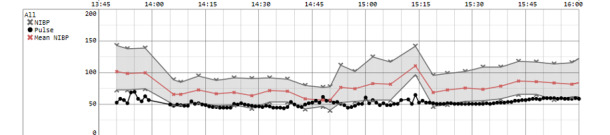
Graph demonstrating the changes in noninvasive blood pressure (NIBP), mean NIBP, and pulse over the duration of the procedure.

Based on the above investigations, diagnoses of hydrocephalus secondary to shunt dysfunction and DCM were made. The shunt revision was prioritized, taking place within the next 3 weeks. The ventriculoatrial shunt was replaced with a ventriculoperitoneal shunt in an uncomplicated procedure. Figure 3 illustrates the changes in blood pressure during the procedure. It is unclear whether precautions were taken with the cervical spine during intubation, such as fiberoptic intubation. A postoperative CT scan confirmed satisfactory ventricular catheter placement and a reduction in ventricular size. The patient was referred to spinal surgery for the management of her DCM following discharge.

**Figure 4:**
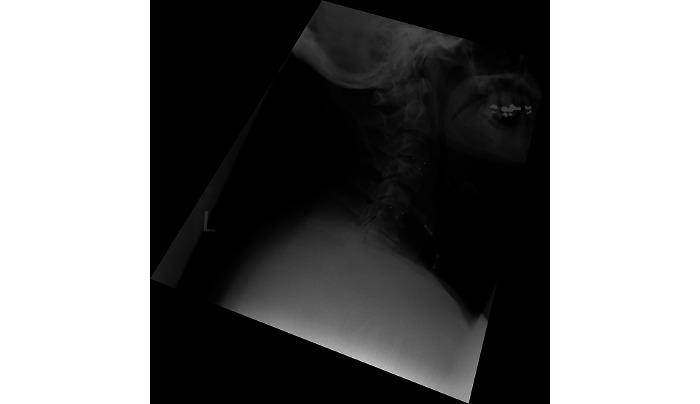
Postoperative x-ray of the cervical spine.

**Figure 5:**
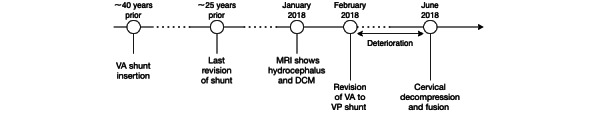
Timeline of events. DCM: degenerative cervical myelopathy; MRI: magnetic resonance imaging; VA: ventriculoatrial; VP: ventriculoperitoneal.

While awaiting an outpatient appointment, the patient experienced a progressive deterioration in her mobility, becoming unable to stand or walk without assistance due to unsteadiness and struggling to use her hands. This led to 2 falls. The patient attended the emergency department. On examination, she had a spastic tetraparesis with urinary urgency and frequency, alongside C7 numbness. Her Modified Japanese Orthopaedic Association (mJOA) score was 9 (2+4+1+2). A CT head scan excluded shunt malfunction. She underwent anterior cervical decompression and fusion (C3/4 and C5/6), with good postoperative recovery. Postoperative imaging is demonstrated in Figure 4. At her 12-month follow up, her mJOA score had recovered to 12 (4+4+2+2). She was able to walk independently, fasten the buttons on her clothes, and peel vegetables but was unable to return to full-time employment. This was unchanged at 24 months. A summary of the timeline of events is illustrated in Figure 5.

### Ethical Considerations

Ethics review board assessment was not required. The patient was given a copy of the manuscript and has consented to publication.

## Discussion

The case describes a patient with both hydrocephalus and DCM. Based on her clinical presentation, the management of the hydrocephalus was prioritized. This coincided with a rapid deterioration in the DCM. Given that DCM is a chronic and progressive condition, it is possible that this was coincidental. However, in early, mild stages of DCM, rapid deterioration is unusual without a trigger. This raises the question as to whether the treatment of hydrocephalus may have inadvertently precipitated a worsening of the DCM. However, it should be noted that other neurological pathology such as hydrocephalus may confound the use of mJOA score as an assessment of DCM severity.

We propose 2 potential mechanisms to explain how shunt surgery might have triggered a deterioration in DCM. The first mechanism is due to general anesthesia, either through neck manipulation for intubation or spinal cord hypoperfusion. Ordinarily, the neck is hyperextended to facilitate intubation. This increases the loading on the spinal cord via stretch but also reduces the spinal canal diameter. For an individual with a normal cervical spine, this has no consequence [4]. However, in patients with cervical stenosis, this could lead to further injury [4] and hypoperfusion [5-7]. Furthermore, anesthetic agents commonly precipitate reductions in blood pressure, which is often most profound during induction and intubation. Chronic hypoperfusion is considered a key feature of DCM, particularly at more advanced stages [8]. Clinical series have shown that DCM can be associated with hypertension that resolves following surgical treatment [9]. This is hypothesized to represent autoregulation [9,10]. Therefore, falls in systolic blood pressure could potentially contribute to the worsening of DCM secondary to hypoperfusion. Generally, anesthetic precautions such as intubation in the neutral position and arterial blood pressure control are taken in patients with DCM; it is unclear how much precaution was taken in this case.

An alternative hypothesis is that the elevated cerebrospinal pressure and volume was protective and that its diversion exacerbated the loading mechanism driving spinal cord injury.

At this stage, which theory or combination of theories explains the deterioration remains uncertain. The perioperative anesthetic management raises concerns but would not explain a body of (albeit low quality) evidence describing similar problems following cerebrospinal diversion, nor importantly a delayed and progressive deterioration of her DCM in the weeks to months after discharge following shunt surgery.

Surgical outcomes for DCM are strongly influenced by baseline disability. Put simply, the goal is to offer surgery when the benefits are known to outweigh the risks, but before there is irreversible damage. While the etiology remains uncertain, it is therefore impossible to suggest whether a different course of action should have been followed. The isolated lower limb presentation of imbalance could very reasonably be associated with the radiologically demonstrated shunt dysfunction. Even in the absence of an alternative diagnosis, for mild levels of impairment, DCM guidelines would suggest surveillance in the first instance. Therefore, in a patient with congenital hydrocephalus for whom shunt revisions had been required and with radiologically confirmed shunt dysfunction, a shunt revision would be the priority. The learning point at this stage is therefore to be aware of the potential risk of DCM deterioration, in order to intervene promptly if necessary.

### Conclusion

This case serves as a reminder that the goal in DCM is to offer surgery when the benefits are known to outweigh the risks, but before there is irreversible damage. While the etiology remains uncertain, given that surgical outcomes for DCM are strongly influenced by baseline disability and symptom duration, surgeons should be alert to the possibility of other surgery, and in particular cerebrospinal diversion, being associated with worsening DCM.

## Abbreviations

CT: computed tomography
DCM: degenerative cervical myelopathy
mJOA: Modified Japanese Orthopaedic Association
MRI: magnetic resonance imaging
